# Avoidant/Restrictive Food Intake Disorder in Children and Adolescents: Nutritional Risk, Diagnostic Boundaries, and Multidisciplinary Care—A Narrative Review

**DOI:** 10.3390/nu18193174

**Published:** 2026-09-26

**Authors:** Marta Tyszkiewicz-Nwafor, Katarzyna Jowik-Krzemińska

**Affiliations:** Department of Adult Psychiatry, Child and Adolescent Psychiatry Ward, Poznan University of Medical Sciences, 27/33 Szpitalna St., 60-572 Poznan, Poland

**Keywords:** avoidant/restrictive food intake disorder, children, adolescents, nutritional deficiencies, growth faltering, pediatric feeding disorder, cognitive-behavioral therapy, family-based treatment

## Abstract

**Background/Objectives:** Avoidant/restrictive food intake disorder (ARFID) involves restrictive or avoidant eating causing clinically significant nutritional, medical, developmental, or psychosocial consequences without being primarily driven by weight- or shape-related psychopathology. This review synthesizes evidence on diagnosis, risk assessment, and treatment of ARFID in children and adolescents aged 0–18 years. **Methods:** We conducted a clinically oriented narrative review informed by a targeted search of English-language publications (1 January 2015–30 June 2026) in PubMed/MEDLINE, Embase, and the Cochrane Library. **Results:** Nutritional deficiencies, growth faltering, pubertal disruption, and psychosocial impairment may occur across the weight spectrum. ARFID presents through overlapping patterns of limited interest in eating, sensory-based avoidance, and fear of aversive consequences. Assessment should integrate maintaining mechanisms, consequences, and alternative diagnoses. Nutritional rehabilitation and psychological intervention form the foundation of care, although controlled evidence remains limited. One randomized trial reported greater weight gain with family-based treatment than with individual psychoeducational motivational therapy in underweight children, without an overall between-group difference in ARFID symptom severity. Pharmacotherapy has no established efficacy and remains adjunctive. **Conclusions:** ARFID requires developmentally informed, multidisciplinary assessment and individualized treatment informed by clinical formulation. Prospective comparative studies are needed to clarify long-term outcomes and treatment selection.

## 1. Introduction

Avoidant/restrictive food intake disorder (ARFID) differs from anorexia nervosa (AN) and bulimia nervosa (BN) in that food restriction is not primarily driven by fear of weight gain or overvaluation of body shape and weight. Instead, ARFID may present with limited interest in eating, avoidance related to the sensory characteristics of food, fear of aversive consequences of eating, or overlapping combinations of these processes. These presentations are clinically useful prototypes rather than exhaustive or mandatory diagnostic categories. ARFID is defined in DSM-5-TR by persistent restrictive or avoidant eating that results in clinically significant nutritional, medical, developmental, or psychosocial consequences [1]. These may include weight loss or faltering growth, nutritional deficiency, dependence on oral nutritional supplements or enteral feeding, and marked psychosocial impairment. The disturbance cannot be better explained by food unavailability or culturally sanctioned practices, and when it occurs alongside a medical condition or another mental disorder, its severity must exceed that ordinarily associated with the co-occurring condition [1].

Over the past decade, ARFID has gained increasing clinical recognition across child and adolescent mental health services, pediatrics, gastroenterology, dietetics and multidisciplinary eating-disorder care. This likely reflects greater awareness of standardized diagnostic criteria and growing recognition of the disorder’s medical, nutritional, and psychosocial burden. ARFID occurs in children and adolescents aged 0–18 years, and its clinical presentation and associated impairments may vary depending on the developmental stage [2,3]. Psychiatric comorbidity and gastrointestinal symptoms may complicate clinical presentation and diagnostic formulation and may contribute to the maintenance of food avoidance [3,4]. ARFID may therefore result in substantial impairment, including disruption of age-appropriate functioning, greater medical compromise, and increased likelihood of hospitalization [3].

Recent research has refined the clinical characterization of ARFID, supported the development of disorder-specific assessment instruments, and generated emerging mechanistic models relevant to case formulation and treatment. Current evidence favors a heterogeneous and dimensional conceptualization in which sensory processing, appetite regulation, fear learning, interoception, neurodevelopmental traits, and environmental responses may interact to shape clinical presentation and course [5,6]. Translating this heterogeneity into clinically actionable decisions remains challenging. Effective assessment must integrate the processes maintaining restriction with its nutritional, medical, developmental, and functional consequences, because neither symptom description nor body weight alone adequately captures clinical severity.

The previous literature includes both broad overviews of ARFID and reviews focused on specific domains, such as epidemiology, gastrointestinal presentations, nutritional complications, assessment, or individual treatment approaches. Building on this literature, the present review provides an updated, pediatric-focused synthesis designed to support multidisciplinary clinical decision-making. Its distinctive contribution is an integrated pediatric framework linking the mechanisms maintaining restriction, the nutritional, medical, developmental, and functional consequences of inadequate intake, and competing diagnostic explanations with current evidence on multidisciplinary treatment. The review also distinguishes ARFID-specific evidence from indirect evidence derived from pediatric feeding disorder, food selectivity, autism, and other restrictive eating disorders.

The aim of this clinically oriented narrative review, informed by a targeted literature search, is to critically synthesize current evidence on the diagnosis, clinical presentation and course, comorbidity, putative mechanisms, medical and nutritional consequences, and treatment of ARFID in children and adolescents. Particular emphasis is placed on clinically relevant diagnostic boundaries, assessment of the mechanisms maintaining restriction and the associated consequences, nutritional and medical risk across the weight spectrum, and the strength and limitations of the available therapeutic evidence.

## 2. Methods

We conducted a clinically oriented narrative review of the literature on ARFID, informed by a targeted literature search, to provide a pragmatic synthesis rather than an exhaustive systematic review. The review covered ARFID nosology and diagnostic boundaries, epidemiology, clinical presentation and course, assessment, nutritional and medical sequelae, comorbidity, putative mechanisms, and treatment. Systematic reviews, meta-analyses, clinical guidelines, and consensus statements were prioritized. Primary studies and clinically relevant conceptual papers were included when they provided additional evidence on specific clinical questions or addressed areas for which higher-quality evidence was limited.

The literature search covered English-language publications from 1 January 2015 through 30 June 2026, including articles available online ahead of print by the latter date. This period was selected to capture contemporary evidence following the introduction and early clinical uptake of the DSM-5 diagnostic criteria for ARFID. Searches were conducted in PubMed/MEDLINE, Embase, and the Cochrane Library using the terms: “avoidant/restrictive food intake disorder (ARFID)”; “ARFID AND selective eating”; “ARFID AND sensory sensitivity”; “food neophobia”; “ARFID neurobiology”; “ARFID complications”; “ARFID treatment”; “ARFID comorbidity,” and “ARFID therapy,” used individually and in combinations. Titles and abstracts of potentially relevant publications, followed when appropriate by full-text review, were assessed independently by both authors; discrepancies were resolved through discussion.

Studies involving children and adolescents were prioritized (0–18 years). Adult and mixed-age studies were considered selectively when pediatric evidence was limited or when their findings were directly relevant to the assessment, nutritional and medical risk, putative mechanisms, or treatment of ARFID in younger populations. Publications were considered when ARFID was the primary focus or when ARFID-specific findings were reported separately within broader feeding- or eating-disorder populations. Case reports and case series were included when they provided clinically relevant information on rare complications or treatment approaches for which stronger evidence was unavailable. Evidence derived from populations without a formal ARFID diagnosis, including picky eating, pediatric feeding disorder, autism-related food selectivity, and selected adult populations, was considered indirect and is identified as such throughout the review.

Sources were selected according to their relevance to the aims of the review and their contribution to multidisciplinary pediatric practice. Evidence was synthesized thematically, with attention to heterogeneity in age groups, study populations, diagnostic definitions, assessment methods, outcome measures, and clinical settings. Because the search was iterative and clinically targeted, formal PRISMA-style counts of identified, screened, and included records were not maintained.

Consistent with the narrative design, no formal study-level risk-of-bias assessment or quantitative synthesis was performed. Findings were interpreted in relation to study design, sample size and population characteristics, methods used to define and assess ARFID, clinical applicability, and consistency across studies. Conclusions were phrased cautiously when evidence was limited, inconsistent, or derived predominantly from lower-level study designs.

## 3. Epidemiology

Epidemiological estimates of ARFID in the pediatric population remain highly variable and differ significantly depending on clinical settings, case identification methods, and the operationalization of diagnostic criteria. In a systematic review focused on pediatrics, reported prevalence estimates varied considerably by setting, ranging from 5% to 22.5% in specialized eating disorder centers and from 32% to 64% in feeding disorder clinics, whereas in samples of children and adolescents not referred to specialized care (nonclinical), estimates ranged from 0.3% to 15.5% [2]. By comparison, in a preregistered meta-analysis comprising 26 studies involving 122,861 participants and combining heterogeneous clinical and nonclinical samples of children and adults, the pooled prevalence of ARFID was 11.14% (95% CI: 8.16–14.50%) in a random-effects model. When a quality-effects model was applied, the estimate decreased to 4.51% (95% CI: 0.70–10.68%), demonstrating the marked sensitivity of pooled prevalence estimates to study quality and methodology and limiting the direct generalizability of this estimate to the pediatric population [7]. Differences between studies also reflect whether ARFID was established based on a diagnostic evaluation or inferred using screening tools or symptom-based definitions, which may identify broader populations than those meeting the full diagnostic criteria [2,7].

Recent population-based findings have begun to address the limited evidence in younger children. In a Swedish study of 645 preschool children, in which parent-reported screening was followed by diagnostic interviews in screen-positive children and a randomly selected group of screen-negative children, the estimated point prevalence of ARFID was 5.9% [8]. These findings extend the epidemiological evidence to early childhood but should be interpreted in the context of the study’s screening-based case-identification procedure and regional sample.

Data on incidence and demographic characteristics derive primarily from surveillance studies. In a Canadian study of children and adolescents aged 5–18 years, the incidence of ARFID was 2.02 per 100,000 (95% CI: 1.76–2.31), the mean age of identified cases was 13.1 years (SD 3.2), and 61.4% were female [3]. In a subsequent surveillance study conducted among individuals aged 5–17 years across the United Kingdom and the Republic of Ireland, the observed annual incidence was 2.79 per 100,000 (95% CI: 2.48–3.13) in the United Kingdom and 0.73 per 100,000 (95% CI: 0.34–1.57) in the Republic of Ireland. The mean age of identified cases in the overall sample was 11.2 years (SD 3.8), and 45.5% were female [9]. These findings suggest that clinically identified ARFID cases include substantial proportions of both females and males. The variation between surveillance systems may reflect differences in case detection, reporting practices, referral pathways, and service organization. These estimates describe cases recognized within healthcare systems rather than true population incidence.

Broader reviews suggest that reported ARFID prevalence estimates may approach those of other eating disorders in some populations and that ARFID can occur across the lifespan. However, directly comparable population data remain limited, which may contribute to variation across published estimates [10].

Evidence regarding the impact of the COVID-19 pandemic on ARFID remains limited. A systematic review identified few relevant studies and found no clear evidence of an increase in ARFID presentations during the pandemic period [11]. In contrast, substantial increases in presentations and hospitalizations were documented for pediatric eating disorders overall, particularly AN [12].

Overall, current prevalence estimates remain highly sensitive to diagnostic definitions, assessment instruments, study populations, and clinical settings [2,7,10]. Much of the available evidence derives from selected clinical or service-based samples, while robust population-level data remain limited. Direct comparisons across studies are therefore difficult, and epidemiological estimates should be interpreted cautiously.

## 4. Diagnostic Framework and Boundaries

The introduction of ARFID in DSM-5 in 2013 established a unified diagnostic framework applicable across childhood, adolescence, and adulthood [13]. At the same time, ARFID remains conceptually rooted in the developmental feeding disorder tradition, which helps explain its position at the intersection of psychiatric, pediatric, and gastroenterological perspectives [14]. The diagnosis replaced the DSM-IV category of “feeding disorder of infancy or early childhood,” which was restricted to younger children [15]. DSM-5-TR retained the overall diagnostic construct introduced in DSM-5 but revised the wording of Criterion A to clarify the role of marked psychosocial impairment [1,13]. DSM-5-TR and ICD-11 both recognize ARFID but differ somewhat in emphasis: DSM-5-TR (code 307.59) more explicitly describes prototypical drivers of avoidance or restriction, whereas ICD-11 (code 6B83) places greater emphasis on insufficient amount and/or variety of intake and the resulting physical or functional impairment [13,16].

In clinical practice, ARFID is not defined by dietary selectivity or the number of accepted foods alone. Diagnosis depends on the persistence of avoidance or restriction and the severity of its nutritional, medical, developmental, and psychosocial consequences in relation to the individual’s developmental stage [1]. However, current diagnostic criteria do not operationalize thresholds for significant weight loss or faltering growth, clinically significant nutritional deficiency, or marked psychosocial impairment. DSM-5-TR also provides no formal severity specifiers for ARFID. Severity must therefore be determined through an integrated clinical assessment rather than a single anthropometric threshold [1].

In pediatric practice, diagnostic uncertainty commonly arises at several clinically important boundaries. ARFID must be differentiated from developmentally normative food selectivity, including food neophobia and picky eating. These behaviors are common in early childhood and may be transient [17,18]. Food neophobia and picky eating are descriptive constructs rather than formal diagnostic entities in DSM-5-TR or ICD-11 and, unlike ARFID, are not defined by clinically significant somatic and/or psychosocial consequences [19].

ARFID must also be considered in relation to pediatric feeding disorder (PFD), an overlapping but conceptually distinct framework. PFD is an interdisciplinary functional construct describing age-inappropriate oral intake associated with dysfunction in at least one of four domains: medical, nutritional, feeding skill, and/or psychosocial [20]. ARFID and PFD are not necessarily mutually exclusive; ARFID is a psychiatric diagnosis, whereas PFD provides a broader functional framework that additionally captures feeding-skill dysfunction [20].

Other important diagnostic boundaries include restriction associated with medical conditions [4] and differentiation from anorexia nervosa or atypical anorexia nervosa [1]; these are discussed in detail in Section 7 and summarized in Table 1.

Overall, these diagnostic boundaries require integrated assessment of chronicity, developmental context, the motivations and mechanisms underlying restriction, and the severity of associated nutritional, medical, and psychosocial consequences [1,4,19,20].

## 5. Clinical Presentation

The clinical presentation of ARFID most commonly organizes around three overlapping profiles: limited interest in food or eating, sensory-based avoidance, and fear of aversive consequences of eating [21]. Limited-interest presentations may involve low appetite, reduced awareness of hunger, early satiety, or limited motivation to eat. Sensory-based avoidance may relate to texture, taste, smell, temperature, appearance, or other characteristics of food. Fear-based avoidance often develops in relation to anticipated choking, vomiting, pain, allergic reactions, or other distressing consequences. These profiles are clinically useful prototypes rather than mutually exclusive diagnostic subtypes and may coexist or vary in prominence within the same individual. A mechanism-oriented formulation therefore focuses on the processes that predominantly maintain restriction, including diminished appetite or interest, sensory hypersensitivity and stimulus avoidance, and fear-related safety behaviors [22].

The clinical course may differ according to the predominant presentation. Sensory-based avoidance and limited interest in eating often develop gradually and may have a longstanding course, whereas fear of aversive consequences may emerge more abruptly after an identifiable event and result in a rapid reduction in intake. Across presentations, severity may be expressed through weight loss or faltering growth, nutritional inadequacy, prolonged or conflictual meals, avoidance of shared eating, and disruption of school, family, and social functioning. Clinically significant impairment is not confined to individuals with low body weight [21].

Prospective evidence suggests that ARFID may follow a persistent course, although individual trajectories vary. In a 2-year study of 100 participants aged 9–23 years with full or subthreshold ARFID, approximately half retained their original diagnosis during follow-up, while 3% experienced diagnostic crossover to AN. Greater baseline severity of sensory sensitivity and limited interest in eating predicted a higher likelihood of ARFID persistence only at 1 year, whereas greater severity of fear of aversive consequences predicted a higher likelihood of remission only at 2 years [23]. These findings suggest that dimensional ARFID presentations may have prognostic relevance, although their generalizability to younger pediatric populations and more diverse samples remains uncertain.

Psychiatric comorbidity is common, with anxiety disorders being the most consistently reported. In pediatric studies, rates of anxiety disorders have ranged from 9.1% to 72% [2]. Obsessive–compulsive and depressive symptoms may also occur as part of the clinical presentation [24]. Recent phenomenological and mechanistic work has drawn particular attention to fear-driven presentations, sometimes termed fear-ARFID. Similarities to panic disorder have been proposed, with heightened interoceptive sensitivity and catastrophic interpretation of bodily sensations representing potential maintaining processes [25].

Neurodevelopmental comorbidity is also prominent. Pediatric studies have reported autism spectrum disorder (ASD) rates ranging from 8.2% to 54.75% [2]. A meta-analysis of 21 studies involving 7442 participants estimated an ASD prevalence of 16.27% in ARFID samples and an ARFID prevalence of 11.41% among autistic individuals [26]. In autistic individuals, sensory processing differences, behavioral rigidity, and restricted preferences may contribute to persistent restrictive eating. However, evidence regarding the mechanisms underlying ARFID and the effectiveness of specific interventions in this population, particularly among older children and adolescents, remains limited [27]. In a single study of gender-diverse adults, ARFID symptoms were associated with autistic traits, ADHD traits, and sensory sensitivities; however, the extent to which these associations apply to pediatric populations remains uncertain, and the absence of a cisgender comparison group precludes conclusions about between-group differences [28].

Gastrointestinal symptoms and disorders of gut–brain interaction (DGBI) are frequently associated with ARFID and may interact bidirectionally with restrictive eating. Gastrointestinal symptoms may trigger or reinforce avoidance, while persistent restriction may worsen nutritional status, gastrointestinal functioning, and psychosocial impairment [4]. ARFID and ARFID-like presentations may therefore occur in patients with gastrointestinal disorders [29]. A recent systematic review of 23 studies involving 6211 participants found substantial heterogeneity in the identification of ARFID in gastrointestinal populations and cautioned that inconsistent application of full diagnostic criteria may overestimate its prevalence. Fear of aversive gastrointestinal consequences was the most consistently reported presentation [30].

Restrictive eating may also become established in the context of organic gastrointestinal disease or medically indicated dietary restriction. In adults with inflammatory bowel disease (IBD), observational findings indicate that ARFID may be present even during inactive disease, with gastrointestinal-specific anxiety identified as a potential predictor [31]. In eosinophilic esophagitis (EoE), food elimination diets may be associated with ARFID. These presentations may require coordinated gastroenterological, dietetic, and psychological care [32].

## 6. Clinical Assessment

Clinical assessment of ARFID integrates dietary and feeding history with evaluation of mental state, developmental context, psychosocial functioning, nutritional status, and medical stability. Establishing the diagnosis requires evidence that avoidant or restrictive eating has resulted in clinically significant nutritional, medical, developmental, or psychosocial consequences, together with careful consideration of alternative explanations [33,34,35,36]. In children and adolescents, assessment should be developmentally informed and multidisciplinary, with specialist involvement determined by the predominant clinical features, mechanisms contributing to restriction, and severity of nutritional and somatic compromise [33,35,36].

A pragmatic formulation can be organized around three core questions: why the patient is not eating adequately, which nutritional, medical, developmental, and psychosocial consequences have emerged, and which alternative diagnoses or exclusion criteria must be considered. Together, these questions provide an organizing framework for assessment, while the relevant differential diagnostic considerations are discussed in the following section [34,35].

Mechanism-oriented assessment evaluates the relative contribution of limited interest in food or eating, sensory-based avoidance, and fear of aversive consequences, recognizing that these mechanisms may overlap and vary across foods, settings, and over time [34,37].

Consequence-oriented assessment evaluates the adequacy and variety of habitual intake, weight and growth trajectories, nutritional deficiencies, and dependence on oral nutritional supplements or enteral feeding. Particular attention should be paid to excluded food groups, principal sources of energy and protein, and dependence on specific brands, textures, preparation methods, supplements, or nutritional support. Nutritional compromise can occur across the weight spectrum, and normal or elevated body mass index does not exclude clinically significant dietary insufficiency. Psychosocial assessment should address participation in school, social and family activities, shared meals, family accommodation, mealtime conflict, and caregiver burden [33,34,36].

Medical risk assessment incorporates longitudinal weight, height, growth velocity, pubertal development, hydration, cardiovascular status, and clinical signs of malnutrition or nutrient deficiency. Laboratory testing should be guided by the duration and severity of restriction, dietary composition, excluded food groups, physical findings, and suspected deficiencies. Values within reference ranges do not necessarily exclude inadequate intake or depleted nutritional reserves. Prolonged or severe restriction, rapid weight loss, and significant undernutrition also require assessment of refeeding risk before nutritional intake is substantially increased [33,34,36,38].

Information should be obtained from both the young person and caregivers. Caregivers may provide important information about early feeding development, changes in accepted foods, growth, meal duration, accommodation, and functioning across settings, whereas direct exploration with the young person may reveal fears, sensory experiences, bodily sensations, avoidance strategies, and psychosocial consequences. Discrepancies between accounts are clinically informative and should be explored [36,37].

Standardized instruments can improve the consistency and dimensional characterization of assessment but do not replace clinical diagnosis. The Pica, ARFID, and Rumination Disorder Interview (PARDI) provides a multi-informant, semi-structured assessment of diagnostic criteria, symptom severity, and the relative contribution of the principal ARFID presentations [37]. Brief questionnaires, including the Nine-Item ARFID Screen (NIAS) and the PARDI-AR-Q, are more suitable for screening and dimensional symptom assessment than for establishing the diagnosis [39,40]. General eating disorder instruments focused primarily on weight- and shape-related psychopathology do not adequately capture the full ARFID construct. Instrument selection should therefore consider age, developmental level, available informants, clinical setting, language version, and validation status, as cutoff values and psychometric performance remain incompletely established across pediatric populations [34,37,39,40].

## 7. Differential Diagnosis

Differential diagnosis requires consideration of developmentally typical food selectivity, pediatric feeding disorder, somatic contributors, particularly gastrointestinal and swallowing-related conditions, anxiety and obsessive–compulsive psychopathology, and weight- or shape-driven eating disorders [17,18,19,20,41,42]. When a medical condition or medically indicated dietary restriction is present, the central question is whether the severity and persistence of food restriction remain proportionate to the underlying condition and its treatment or have developed into a clinically maladaptive eating disturbance [42]. Persistent restriction that exceeds what would be expected from the underlying condition or results in clinically significant nutritional or psychosocial impairment supports the latter interpretation [42].

A similar distinction is required in anxiety disorders and obsessive–compulsive disorder, including specific phobias and emetophobia. Assessment should determine whether reduced intake occurs only as a consequence of the primary anxiety process or represents a persistent avoidant or restrictive eating pattern that independently meets diagnostic criteria for ARFID [41,43]. The presence of anxiety, obsessive–compulsive symptoms, or a neurodevelopmental condition does not preclude ARFID when the eating disturbance exceeds what would ordinarily be expected from the co-occurring condition and results in clinically significant nutritional, medical, developmental, or psychosocial consequences [41].

Differentiation from AN and atypical AN is particularly important. A recent systematic review and meta-analysis of 22 comparative studies found that, compared with young people with AN, those with ARFID were younger, included a higher proportion of males, had higher odds of anxiety and lower odds of depressive disorders, and were less frequently hospitalized. Comparisons with BN similarly showed younger age, a higher proportion of males, and lower odds of depressive disorders [44]. These group-level differences, including differences in psychiatric comorbid symptom severity, may inform clinical expectations but cannot distinguish the disorders in an individual patient [44,45].

Clinical evaluation therefore requires direct exploration of fear of weight gain, overvaluation of weight or shape, compensatory behaviors, and other motivations for restriction [41,46]. ARFID-related and shape/weight-related motivations may overlap in some patients [46]. Conversely, concern about low weight or the somatic consequences of malnutrition should not in itself be equated with weight- or shape-driven restriction.

In diagnostically ambiguous presentations, ARFID and AN or atypical AN may need to be considered in parallel. The clinical formulation should explicitly document the presence or absence of weight- and shape-related motivation, compensatory behaviors, eating-related fear, sensory avoidance, and limited interest in eating. When uncertainty persists, longitudinal reassessment may help clarify whether weight- or shape-related motivations emerge or whether ARFID-related motivations remain predominant [41,46].

The principal clinical features relevant to the differential diagnosis of avoidant and restrictive eating in children and adolescents are summarized in Table 1.

Finally, longitudinal documentation of ARFID symptom severity and its medical, nutritional, developmental, and functional consequences supports monitoring of treatment response. Where feasible, standardized outcome measures may improve consistency and comparability across clinical settings and over time [47]. Baseline and follow-up assessment may also support evaluation of treatment outcomes and potential predictors of response [48]. The following section summarizes the medical, nutritional, and developmental sequelae relevant to clinical monitoring and decisions regarding level of care.

## 8. Clinical Sequelae

In children and adolescents, ARFID may lead to medical, nutritional, and developmental sequelae resulting from insufficient energy intake, markedly restricted dietary variety, or both [49,50]. A systematic review of 132 studies identified nutritional deficiencies, reduced bone mineral density, and cardiovascular abnormalities, including bradycardia and hypotension in a subset of patients, and highlighted the need to monitor growth and pubertal development [49]. Clinically significant complications may occur across the weight spectrum; therefore, medical and nutritional risk cannot be inferred from body mass index alone [49] (Table 2).

Micronutrient deficiencies are particularly relevant when dietary variety is severely restricted, even when total energy intake is sufficient to maintain body weight. A qualitative review of case reports and case series involving children and adolescents with autism or the broad autism phenotype and restrictive eating consistent with ARFID identified scurvy as the most frequently reported deficiency-related complication, often in patients whose body weight remained within the expected range [58]. These findings illustrate how a narrowly restricted diet may produce clinically significant and potentially severe deficiencies despite apparently adequate weight status [49,58].

When restrictive eating begins before or during puberty, prolonged nutritional insufficiency may disrupt both linear growth and pubertal progression. A systematic review of growth outcomes in restrictive eating disorders indicated that catch-up growth was not universal and may be influenced by age at onset, duration of illness, and the degree of nutritional recovery achieved during treatment [62]. Downward crossing of growth percentiles is therefore clinically meaningful even when current weight or height remains within population reference ranges [55].

Prolonged restriction may also compromise skeletal development during a critical period of bone mass accrual. Available comparative data indicate low bone mineral density in underweight young people with ARFID, supporting explicit consideration of bone health in this population [63]. Risk may be particularly relevant when restrictive intake coexists with impaired linear growth, delayed pubertal development, or prolonged nutritional inadequacy [50,62,63]. In the broader literature on restrictive eating disorders in young people, impaired growth, pubertal disruption, and reduced bone mass may have longer-term developmental consequences [62]. Cardiovascular abnormalities related to malnutrition are often substantially reversible with nutritional rehabilitation, although the medical risk profile associated with ARFID may differ in some respects from that observed in AN [51].

Rare but potentially severe or irreversible complications have been described at the extreme end of the clinical spectrum. Nutritional blindness has been reported in ARFID and may be especially difficult to recognize in younger children and those with ASD [59], while Wernicke encephalopathy has been described in an 8-year-old child whose restrictive eating was driven by fear of choking [60]. Although these reports describe uncommon presentations and derive from lower-level evidence, they demonstrate the potential severity of prolonged restriction, highly limited dietary variety, and delayed recognition. Consistent with these observations, the Society for Adolescent Health and Medicine has emphasized that medical complications in adolescents and young adults with ARFID may be comparable in severity to those observed in anorexia nervosa [52].

The burden of ARFID extends beyond somatic and nutritional morbidity. Psychiatric comorbidity, particularly anxiety disorders and, in a subset of patients, mood disorders, is common and may increase treatment complexity [67,68]. A pediatric study indicated reduced health-related quality of life, including poorer psychosocial and school functioning [64]. Direct ARFID-specific evidence on cognitive outcomes remains limited. The broader pediatric nutrition literature supports an important role for adequate nutrition in cognitive development; however, the extent to which attention or concentration difficulties in ARFID are directly attributable to undernutrition or micronutrient deficiency remains uncertain [69]. Parent-report and qualitative studies further describe substantial caregiver burden, mealtime stress, and extensive adaptation of family routines to accommodate restrictive eating [65,66].

Together, these medical, nutritional, developmental, and functional consequences underscore the need to evaluate severity across multiple domains rather than relying on body weight alone. They also provide the clinical context for the mechanistic models of ARFID phenotypes and maintaining processes discussed in the following section.

## 9. Mechanisms and Neurobiology

ARFID is increasingly conceptualized as a heterogeneous disorder in which partially distinct but overlapping mechanisms may lead to dietary restriction or avoidance. A proposed three-domain neurobiological model links alterations in sensory perception, homeostatic appetite regulation, and negative-valence systems with the three prototypical ARFID presentations: sensory sensitivity, limited interest in eating, and fear of aversive consequences [70]. This model is based in part on indirect evidence from studies involving adult populations and mixed-age groups, which are considered potentially relevant to pediatric ARFID due to the phenomenological similarity of sensory, appetitive, and anxiety symptoms across age groups, rather than on direct confirmation in a pediatric population. Within this framework, negative valence refers to fear responsiveness and aversive processing that may strengthen avoidance in eating-related situations. The model provides a dimensional framework for organizing clinical heterogeneity rather than a set of discrete biological subtypes.

The broader literature on children’s feeding difficulties highlights interactions between individual predispositions, learning processes, and the feeding environment [71]. Individual vulnerabilities, including sensory sensitivity and reduced responsivity to hunger signals, may interact with caregiver responses, mealtime interactions, and feeding practices. Responsive support and pressure to eat may have different effects depending on the child’s predominant vulnerabilities. Clinical presentation and course may therefore emerge from reciprocal interactions between individual predispositions and the feeding environment rather than from a single causal pathway [70,71].

Neurodevelopmental traits may form part of this broader vulnerability profile. In children and adolescents with ASD, atypical sensory processing and behavioral rigidity have been associated with restrictive and atypical eating patterns, including presentations consistent with ARFID [72]. Difficulties with cognitive flexibility and tolerance of uncertainty may further influence food acceptance and adaptation to changing eating demands. In some patients, ARFID may therefore be embedded within a broader developmental phenotype, while still requiring assessment of the specific mechanisms and consequences of the eating disturbance.

Mechanistic hypotheses have also incorporated gut–brain signaling and learning-based processes. One conceptual model proposes that restricted intake and limited dietary variety may alter gut microbiota diversity and microbial metabolites, which could in turn influence appetite regulation, interoceptive processing, stress responsivity, and the perception of food-related signals [73].

This hypothesis is based largely on studies of the microbiota in other conditions associated with dietary restriction, rather than directly in the ARFID population; the direction and clinical significance of these associations have not been established, and it remains unclear whether changes in the microbiome contribute to ARFID symptoms, result from restricted eating, or reflect bidirectional processes. In parallel, preclinical conditioned taste-aversion paradigms have been proposed as a framework for understanding persistent avoidance in pediatric feeding disorder and ARFID, particularly when food restriction follows an aversive eating-related or medical experience. Evidence from these preclinical models is indirect with regard to pediatric ARFID and is relevant primarily because it provides a mechanistic framework for aversion learning rather than because it has been empirically validated in children with ARFID; these models emphasize learned fear and aversion, inhibitory learning, reconsolidation, and extinction [74].

The biological basis of ARFID remains substantially less characterized than that of anorexia nervosa. ARFID-specific neuroimaging studies [75,76], together with broader neuroimaging work in early-onset restrictive eating disorders [77], have identified structural and functional correlates involving frontal cortical regions, food-cue responsivity, attention, multisensory integration, and neural systems implicated in aversive processing. These findings provide indirect evidence with regard to pediatric ARFID; their potential relevance derives from overlap in clinical phenotypes related to dietary restriction across age and diagnostic groups rather than from studies conducted directly in representative pediatric ARFID samples. These findings can be interpreted within the three-domain model, particularly in relation to sensory processing, appetite and reward responsivity, and negative-valence systems, but they are not yet sufficient to confirm, refine, or biologically validate the proposed framework. Current neurobiological findings therefore complement clinical formulation but do not support routine biomarker-based diagnosis, phenotyping, or treatment selection.

From a transdiagnostic perspective, altered processing of gastrointestinal signals and maladaptive interpretation of visceral sensations may also contribute to symptom maintenance [78]. The evidence in this area comes primarily from studies on anxiety disorders and functional gastrointestinal disorders in adults, and its relevance to ARFID remains indirect, stemming from the similarity in the mechanism of visceral hypersensitivity that underpins avoidance behaviors, rather than from studies conducted directly in the ARFID population. This framework may be particularly relevant to fear-based ARFID, in which heightened sensitivity to gastrointestinal or swallowing-related sensations may reinforce avoidance. Interoceptive processes may also be relevant to limited-interest presentations, although direct ARFID-specific evidence remains limited [78]. However, the role of interoception is likely to vary across patients and should be evaluated within an individualized clinical formulation rather than assumed from the diagnostic label alone.

Taken together, current models support a dimensional and mechanism-informed understanding of ARFID. Sensory processing, appetite regulation, fear learning, interoception, neurodevelopmental traits, gastrointestinal signaling, and the feeding environment may contribute in different combinations across individuals and across stages of illness. However, a substantial proportion of the available evidence remains indirect, deriving from adult or mixed-age populations, neuroimaging studies, and preclinical models. The available biological evidence remains preliminary and does not yet provide biomarkers useful in clinical practice. These models offer a useful, though still hypothetical, framework for clinical formulation and for generating hypotheses about how interventions might target the processes most directly underpinning restriction or avoidance, rather than an empirically validated strategy for treatment selection.

## 10. Treatment

### 10.1. General Principles

Treatment of ARFID in children and adolescents requires coordinated medical, nutritional, psychological, and, when indicated, feeding-skill interventions [14,20,53,56]. Core management principles include early identification, assessment of medical and nutritional risk, restoration of adequate intake, reduction in avoidant or restrictive eating, and multidisciplinary care [53,56]. Primary care clinicians and pediatricians play an important role in early recognition, initial medical assessment, coordination of care, and referral to appropriate specialist services [53]. Treatment goals should address medical and nutritional status, eating behavior, psychological symptoms, developmental needs, and functional impairment and should be reviewed as the clinical presentation changes [14,20,53,56].

### 10.2. Nutritional Rehabilitation

Nutritional management aims to restore adequate energy and nutrient intake while gradually increasing dietary variety and flexibility. Individualized strategies may use accepted foods as a starting point and incorporate food chaining where appropriate. Dietetic assessment should consider the amount and variety of habitual intake, excluded food groups, principal sources of energy and protein, reliance on particular brands or methods of preparation, and the use of fortified products, supplements, oral nutritional supplements, or enteral nutrition [61]. Restoration of nutritional adequacy may need to precede broader dietary expansion, particularly when rapid weight loss, growth faltering, medical instability, or severe nutrient deficiency is present [56,61].

Micronutrient assessment and management should be guided by the dietary pattern, clinical manifestations, and laboratory findings rather than inferred from body weight alone. Marked exclusion of specific food groups may be associated with clinically significant deficiencies, including deficiencies of vitamin C, thiamine, vitamin A, iron, folate, vitamin B12, vitamin D, calcium, or zinc [58,59,61]. Identified or strongly suspected deficiencies require targeted dietary fortification or supplementation, with the route, dose, and urgency determined by clinical severity [59,60,61]. When a severe deficiency associated with serious or potentially irreversible consequences is suspected, particularly thiamine deficiency, treatment may need to begin before laboratory confirmation is complete [59,60]. Nutrient replacement should be accompanied by efforts to improve the long-term adequacy and diversity of the diet rather than relying indefinitely on supplementation as the sole intervention [59,61].

### 10.3. Level of Care and Feeding Support

Treatment should be provided in the least restrictive setting that can safely address the patient’s medical, nutritional, psychiatric, developmental, and functional needs, with escalation when these needs can no longer be managed adequately in outpatient care or when medical instability develops [53,56]. When hospitalization is required, immediate priorities include medical stabilization, safe nutritional rehabilitation, monitoring for complications of malnutrition and refeeding, and formulation of an individualized multidisciplinary treatment plan [54,56,57]. ARFID-specific inpatient pathways may reduce reliance on the unmodified application of restrictive eating disorder protocols [57].

Oral nutritional rehabilitation is preferred when swallowing is safe and adequate nutritional intake can be achieved by mouth [56]. Enteral feeding may nevertheless be required when oral intake remains insufficient to achieve medical stabilization, prevent further nutritional deterioration, or permit safe nutritional rehabilitation [56,57]. Evidence directly comparing oral and enteral nutritional rehabilitation strategies in pediatric ARFID remains limited [56]. Decisions regarding enteral nutrition should be based primarily on medical and nutritional necessity while also considering the patient’s clinical presentation and ability to participate in oral nutritional rehabilitation [56,57]. When enteral support is used, it should be integrated thoughtfully into the multidisciplinary treatment plan [56,57].

Evidence for oral nutritional supplements derives from randomized trials in underweight children with picky eating rather than from trials conducted specifically in children with ARFID. Oral nutritional supplements combined with dietetic counseling may support weight gain and improve selected growth outcomes, although evidence regarding longer-term outcomes remains limited [79]. They should be used as adjuncts rather than substitutes for interventions aimed at improving the adequacy and variety of the habitual diet [61,79].

Some children require specific assessment of feeding or swallowing skills, particularly when dysphagia, impaired chewing, oral-motor dysfunction, difficulty progressing to developmentally appropriate textures, or co-occurring pediatric feeding disorder is suspected. When ARFID and feeding-skill dysfunction coexist, both domains should be addressed within the multidisciplinary treatment plan [14,20].

### 10.4. Psychological Interventions

Psychological treatment is a central component of ARFID care, but the evidence base remains limited [47,80]. A recent systematic review of 40 studies, including 17 studies in a meta-analysis, found significant pre-to-post improvements in ARFID psychopathology, body weight, anxiety, and depressive symptoms following psychological interventions in adolescents and adults. However, the available evidence consisted predominantly of case reports and case series, with very few quasi-experimental or randomized studies. These findings therefore provide preliminary support for psychological interventions but do not permit firm conclusions regarding their comparative or causal efficacy [80]. Common elements across evaluated interventions include psychoeducation, establishment of regular eating, food exposure, strategies targeting anxiety and avoidance, and caregiver involvement [47,80]. Treatment planning should consider the predominant maintaining mechanism, developmental stage, and broader clinical context, although evidence supporting formal matching of specific interventions to particular ARFID presentations remains limited [47,81].

Cognitive-behavioral therapy for ARFID, or CBT-AR, is among the most clearly articulated ARFID-specific approaches. It was developed for medically stable patients aged 10 years and older and is typically delivered over approximately 20–30 sessions in four stages, using either an individual or family-supported format [81]. Preliminary uncontrolled studies have reported improvements in ARFID symptoms and dietary variety, with weight gain among underweight participants, but adequately controlled evidence remains limited [47,80]. CBT-AR targets three prototypic motivations for restriction—sensory sensitivity, lack of interest or low appetite, and fear of aversive consequences—through presentation-specific treatment components [22,81]. For presentations predominantly maintained by fear of aversive consequences, treatment may include graduated exposure to feared foods and eating situations, reduction in safety behaviors, and exposure to feared bodily sensations. Such adaptations draw on principles used in the treatment of anxiety and panic disorders, although their ARFID-specific efficacy requires further investigation [25,81].

In autistic children and young people with severe food selectivity, feeding difficulties, or co-occurring ARFID symptoms, behavioral interventions may increase food acceptance and reduce challenging mealtime behaviors [72]. In autistic children under six years of age with food selectivity, applied behavior analysis-based strategies, alone or combined with other approaches, have been the most frequently evaluated interventions for improving food acceptance and reducing challenging mealtime behavior, although that literature explicitly excludes children with diagnosed eating disorders [82]. These approaches may include reinforcement-based strategies, shaping, modeling, stimulus fading, and graduated exposure. Adaptation to the individual’s communication style, sensory profile, and developmental level may be required [72]. However, the evidence remains heterogeneous and derives predominantly from small studies of food selectivity and feeding difficulties, particularly in younger children, rather than from controlled trials conducted specifically in autistic children and adolescents with ARFID [72,82]. Improvements in acceptance of particular foods or mealtime behavior should not automatically be interpreted as broader improvements in nutritional adequacy, dietary variety, or ARFID psychopathology.

### 10.5. Family-Based and Parent-Focused Interventions

Family-based treatment for ARFID adapts the core principles of family-based treatment to empower caregivers to take an active role in increasing the amount and/or variety of food consumed. The immediate treatment emphasis may differ depending on whether restriction is maintained primarily by sensory sensitivity, fear of aversive consequences, or limited interest in eating. Caregiver involvement may include supporting meals, promoting changes in dietary volume or variety, and challenging longstanding accommodation of avoidant eating. The initial description of FBT-ARFID was based on three clinical cases and therefore established the treatment model rather than its efficacy [83].

SPACE-ARFID is a parent-based intervention focused on parental responses to ARFID symptoms and the reduction in family accommodation. In a small uncontrolled pilot study, the intervention was feasible and acceptable and was associated with reductions in ARFID symptom severity, impairment, and family accommodation, as well as increased food-related flexibility [84]. These findings are preliminary because the study did not include a control group. More broadly, the optimal selection, sequencing, intensity, and duration of family-based and parent-focused interventions remain uncertain [47,83,84].

In a randomized clinical trial, 98 underweight children aged 6–12 years were assigned to 14 sessions over four months of telehealth FBT-ARFID or individual psychoeducational motivational therapy. FBT-ARFID produced a significantly greater increase in percent estimated body weight by the end of treatment. Both treatments were associated with improvement in ARFID symptom severity, with no significant overall between-group differences in ARFID symptom severity or other related ARFID symptoms; the weight gain observed in the FBT-ARFID arm was not paralleled in the individual treatment arm [85]. These findings support FBT-ARFID for promoting weight gain in low-weight school-aged children. Their generalizability to adolescents, patients without low weight, and patients requiring intensive medical or feeding support remains uncertain [85].

### 10.6. Pharmacotherapy and Comorbidity Management

No medication has established efficacy for ARFID itself. At the time of the 2023 WFSBP update, no randomized controlled trial of pharmacotherapy for ARFID had been published [86]. When used in the context of ARFID treatment, pharmacotherapy is therefore adjunctive and generally off-label, targeting comorbid anxiety or depressive symptoms or selected clinical barriers to nutritional rehabilitation, such as low appetite, rather than the core disorder itself [86]. The 2023 WFSBP update concluded that the available evidence was descriptive and derived predominantly from case reports, case series, and retrospective studies, frequently involving polypharmacy and concurrent psychological and nutritional interventions, thereby limiting causal interpretation. The guideline assigned low evidence (LoE C2) and weak recommendations (GoR 3) to mirtazapine, selective serotonin reuptake inhibitors, and olanzapine [86]. A subsequent retrospective study of 87 hospitalized children and adolescents found that mirtazapine treatment was associated with a shorter hospital stay, fewer nasogastric-feeding days, and a faster rate of weight gain; however, its nonrandomized design does not establish medication efficacy [87]. When medication is considered, selection should be guided by the target symptoms, comorbidities, medical and nutritional status, and adverse-effect profile, as there is insufficient evidence to support differential pharmacological treatment according to ARFID presentation [86,87,88]. Pharmacotherapy should remain embedded within a comprehensive treatment plan that includes medical monitoring, nutritional rehabilitation, and psychological intervention [86,87,88].

Psychiatric comorbidities and gastrointestinal symptoms may interact with avoidant or restrictive eating, contribute to the persistence of food avoidance, and complicate nutritional and psychological treatment [4,67]. Anxiety and depressive symptoms, neurodevelopmental conditions such as ASD and ADHD, and gastrointestinal complaints therefore require concurrent assessment and appropriate management [4,68]. These conditions should be addressed as parallel and interacting treatment targets within a coherent multidisciplinary plan rather than treated in isolation from the eating disturbance.

As summarized in Table 3, current interventions are clinically promising, but the evidence base remains constrained by the small number of randomized trials, heterogeneous treatment models, and inconsistent or insufficiently validated outcome measures [47,80,85]. Evidence remains sparse or indirect for dietetic and feeding-skill interventions and for the long-term maintenance of treatment gains, while comparative evidence supporting the selection of interventions according to clinical presentation remains limited [20,47,56,80]. Accordingly, tailoring interventions to the mechanism underpinning food restriction should currently be regarded as a clinical hypothesis requiring further empirical verification rather than as an established therapeutic algorithm.

## 11. Limitations and Future Directions

This review should be interpreted in the context of both design-related limitations and constraints inherent to the current ARFID literature. As a clinically oriented narrative synthesis, it was intended to provide a pragmatic overview of evidence relevant to clinical practice rather than a comprehensive systematic evaluation of all available studies. Restriction to English-language publications may have resulted in the omission of relevant evidence. No formal study-level risk-of-bias assessment was performed, limiting the certainty with which the methodological quality of individual studies could be evaluated. Evidence published after 30 June 2026 was not included. A further limitation is that, in several areas where direct pediatric ARFID evidence is sparse, the review necessarily draws on indirect evidence from related feeding, neurodevelopmental, gastrointestinal, or adult populations. In addition, some of the monitoring and escalation considerations summarized in Table 2 are derived from consensus-based guidance and the broader pediatric restrictive eating disorder literature rather than from ARFID-specific empirical studies. The conclusions are therefore shaped by both the scope of the selected literature and the quality of the underlying evidence base.

A major challenge is the marked heterogeneity of ARFID research, including variation in diagnostic operationalization, assessment instruments, study populations, outcome definitions, and care settings. Differences between clinical and nonclinical samples and between pediatric and mixed-age populations limit direct comparison and generalizability across studies [47,89,90]. Interpretation is further complicated by uncertainty at the boundaries of the ARFID construct, particularly in relation to restriction associated with medical conditions and pediatric feeding disorder, as well as by inconsistent terminology across disciplines [4,20]. The limited geographic and cultural diversity of available samples further constrains the applicability of findings across populations and settings [2,90].

Five broad evidence gaps remain. First, although emerging prospective evidence has begun to characterize short-term course [23] and treatment studies have begun to identify predictors of outcome [48], the natural history and long-term prognosis of ARFID remain insufficiently understood. Large, developmentally stratified longitudinal cohorts are needed to examine persistence, remission, diagnostic crossover, relapse, and functional outcomes across key transitions from childhood through young adulthood [23,90].

Second, nutritional and feeding research lacks standardized approaches to dietary assessment and micronutrient screening, clear evidence-based indications for targeted supplementation, oral nutritional supplements, and enteral nutritional support, and robust ARFID-specific evaluation of dietetic and feeding-skill interventions [20,56,61,79]. Long-term data are also needed to determine how the timing and adequacy of nutritional rehabilitation influence linear growth, pubertal development, and bone health [62,63].

Third, assessment and diagnosis remain limited by incomplete validation of instruments across populations and clinical settings, together with the absence of harmonized measures of symptom change and treatment response [39,40,91].

Fourth, mechanistic understanding remains preliminary: neurobiological evidence remains limited, while gut–brain and microbiome models remain largely hypothesis-generating rather than clinically actionable [70,73,75,76].

Fifth, comparative treatment evidence remains insufficient to determine which interventions and treatment components work best, for whom, at which stage of illness, and for which predominant maintaining mechanisms. The field continues to rely on relatively few randomized trials, varied intervention formats and endpoints, and limited evidence on maintenance of improvement, relapse, and presentation-specific treatment selection [80,85,90,92].

Future research should prioritize prospective cohort studies and comparative trials using clearly defined inclusion criteria, standardized diagnostic procedures, and core outcome sets that capture nutritional, medical, psychological, developmental, and functional outcomes [47,90]. Treatment studies should distinguish restoration of energy intake and weight from improvement in dietary variety, reduction in fear and avoidance, greater psychosocial participation, and decreased dependence on oral supplementation or enteral feeding [47,92]. Studies should include culturally, geographically, and clinically diverse populations and reflect the full range of settings in which ARFID is identified and managed [2,90]. Pragmatic and implementation-oriented research is also needed to evaluate stepped-care models, referral pathways, multidisciplinary treatment configurations, and criteria for escalation and de-escalation of care in nonspecialist settings, including primary care, pediatric, and gastroenterology services, using clinically meaningful somatic and functional outcomes [53,90,93].

## 12. Conclusions

ARFID is a clinically significant and heterogeneous disorder whose severity cannot be inferred from body weight, dietary variety, or symptom presentation alone. Avoidant or restrictive eating may result in nutritional deficiency, impaired growth and development, dependence on nutritional support, and marked psychosocial impairment across the weight spectrum. Accurate recognition therefore requires an interdisciplinary perspective and assessment of three linked domains: the mechanisms maintaining restriction, its nutritional, medical, developmental, and psychosocial consequences, and competing diagnostic explanations. Longitudinal growth and weight trajectories, dietary adequacy, micronutrient risk, medical stability, psychosocial functioning, family accommodation, and caregiver burden should be considered together, because no single symptom or anthropometric measure adequately captures clinical severity.

Treatment should be developmentally informed, multidisciplinary, and anchored in explicit nutritional, medical, psychological, developmental, and functional targets. Nutritional rehabilitation and psychological intervention form the foundation of care, while selected patients may additionally require feeding- or swallowing-skill interventions. Pharmacotherapy remains adjunctive and should target comorbid symptoms or selected clinical barriers to treatment rather than ARFID itself. Despite emerging prospective and randomized evidence, major uncertainties remain regarding long-term prognosis, standardized outcome measurement, optimal nutritional and feeding interventions, maintenance of treatment gains, and comparative treatment effectiveness. Current evidence supports early recognition, integrated assessment, and coordinated multidisciplinary care but does not yet establish a single treatment algorithm or reliable presentation-specific treatment selection. Primary care clinicians and pediatricians remain central to early identification, assessment of medical and nutritional risk, and timely referral to appropriate services.

## Figures and Tables

**Table 1:** Differential diagnosis and exclusion criteria for avoidant and restrictive eating in children and adolescents.

Clinical Presentation	Predominant Basis or Driver of Restriction	Clinical Consequences	Key Distinguishing Features
Avoidant/restrictive food intake disorder (ARFID)	Limited interest in eating, sensory sensitivity, and/or fear of aversive consequences	Weight loss or growth faltering, nutritional deficiency, dependence on nutritional support, and/or marked psychosocial impairment	Restriction is not primarily driven by weight or shape concerns; clinically significant consequences are required. ARFID is not diagnosed when restriction occurs exclusively during the course of AN or BN
Developmentally typical picky eating	Food neophobia and preferences for familiar foods, tastes, or textures	Usually no clinically significant nutritional, developmental, or psychosocial impairment	A descriptive construct rather than a formal diagnosis; common and often transient in early childhood, with generally preserved growth and functioning
Pediatric feeding disorder (PFD)	Dysfunction in medical, nutritional, feeding-skill, and/or psychosocial domains	May include inadequate intake, impaired feeding skills, aspiration risk, nutritional compromise, or caregiver burden	Interdisciplinary functional construct; feeding-skill dysfunction is particularly relevant; PFD and ARFID may coexist
Anorexia nervosa or atypical anorexia nervosa	Fear of weight gain, pursuit of thinness, or overvaluation of weight or shape	Weight loss, malnutrition, medical instability, growth disruption, and functional impairment	Weight- and shape-related psychopathology is central, although it may initially be minimized or concealed
Restriction associated with a medical condition	Pain, nausea, dysphagia, allergy, gastrointestinal symptoms, or medically indicated dietary restriction	Consequences may result from the illness, reduced intake, or both	ARFID should be considered when restriction is disproportionate to the underlying condition, persists despite treatment, or extends beyond medically necessary limitations
Restriction associated with anxiety or OCD	Phobic fear of choking, vomiting, or contamination; obsessional concerns about food safety or preparation	Variable nutritional and psychosocial impairment	ARFID when restriction exceeds anxiety or OCD alone and causes clinically significant consequences
Autism-related food selectivity	Sensory sensitivity, rigidity, insistence on sameness, and restricted preferences	Long-standing narrow diet; nutritional risk may occur at normal weight	Autism does not preclude ARFID; the diagnosis applies when restriction and its nutritional, medical, developmental, or psychosocial consequences exceed those ordinarily expected from autism alone. Consider co-occurring PFD when feeding-skill dysfunction is present.
Food unavailability or culturally sanctioned practices	Limited food access; culturally or religiously sanctioned fasting or dietary practices	Possible inadequate intake or weight loss	DSM-5-TR exclusion: ARFID is not diagnosed when restriction is fully explained by these circumstances

These presentations are not always mutually exclusive. Longitudinal reassessment may be required when the motivation for restriction is unclear or when several mechanisms coexist. Abbreviations: AN, anorexia nervosa; ARFID, avoidant/restrictive food intake disorder; BN, bulimia nervosa; OCD, obsessive–compulsive disorder; PFD, pediatric feeding disorder.

**Table 2:** Clinical risk domains, suggested monitoring, and considerations for escalation of care in pediatric avoidant/restrictive food intake disorder.

Risk Domain	Key Clinical Indicators	Suggested Monitoring	Considerations for Escalation of Care
Physiological instability	Syncope, bradycardia, hypotension, orthostasis, hypothermia, dehydration, or suspected arrhythmia	Vital signs, orthostatic measurements, temperature, hydration status, and ECG when indicated	Urgent medical assessment; inpatient stabilization when abnormalities are clinically significant [49,51,52,53,54].
Inadequate intake, nutritional dependence, or rapid weight loss	Progressive restriction in amount or variety; limited meals or portions; inadequate energy, protein, or fluid intake; rapid weight loss or growth faltering; increasing dependence on fortified foods, oral supplements, or enteral feeding	Weight, height, growth trajectory, recent weight change, dietary and fluid intake, excluded food groups, principal energy and protein sources, and current nutritional support	Higher-intensity or inpatient care when nutritional or hydration needs cannot be met safely, or weight loss or growth compromise progresses [49,53,54,55,56,57].
Micronutrient deficiency	Extremely narrow diet; exclusion of major food groups; dependence on a limited range of foods, brands, textures, or preparation methods; fatigue, pallor, bruising, mucocutaneous, neurologic, or visual symptoms	Dietary history; blood count, ferritin, vitamin B12, folate, and 25(OH)D; vitamin C, vitamin A, zinc, and thiamine when indicated	Urgent assessment for severe or potentially irreversible complications; suspected thiamine deficiency requires treatment without awaiting laboratory confirmation [49,58,59,60,61].
Refeeding risk	Prolonged or severe restriction; minimal intake; rapid or substantial weight loss; very low weight; previous refeeding complications	Phosphate, potassium, magnesium; fluid balance; cardiac and neurological status	Inpatient nutritional rehabilitation with electrolyte monitoring and supplementation when risk is high [52,53,54,56].
Growth, puberty, and bone health	Crossing growth percentiles; reduced height velocity; delayed puberty; menstrual disturbance; prolonged undernutrition; bone pain or low-trauma fractures	Longitudinal growth trajectory, height velocity, pubertal stage, menstrual and fracture history, 25(OH)D, and DXA when indicated	Specialist assessment; urgent evaluation of low-trauma fractures; higher-intensity care when growth, pubertal, or skeletal abnormalities progress [38,50,55,62,63].
Gastrointestinal, swallowing, or feeding-skill risk	Persistent vomiting, dysphagia, choking, severe pain, dehydration, impaired chewing, or inability to manage developmentally appropriate textures	Hydration, intake, weight trajectory, mealtime observation, and targeted gastrointestinal, swallowing, oral-motor, or feeding-skill assessment	Urgent assessment when nutrition or hydration cannot be maintained or when aspiration or significant swallowing dysfunction is suspected [14,20,53,56].
Marked functional impairment	School absence; inability to eat outside the home; avoidance of shared meals; severe social or family disruption; substantial caregiver burden	School, social, and family functioning; age-appropriate participation; caregiver accommodation; mealtime conflict; treatment response	Higher-intensity multidisciplinary care when impairment persists or worsens despite appropriately delivered outpatient treatment [33,34,36,53,56,64,65,66].

Decisions regarding level of care should be individualized according to medical stability, developmental status, symptom trajectory, treatment response, available services, and local admission criteria. This table is not a substitute for comprehensive medical assessment. Abbreviations: 25(OH)D, 25-hydroxyvitamin D; DXA, dual-energy X-ray absorptiometry; ECG, electrocardiogram. The monitoring and escalation considerations presented in this table synthesize ARFID-specific observational evidence together with pediatric eating disorder guidelines, consensus recommendations, and inpatient pathway literature. They should not be interpreted as empirically validated ARFID-specific thresholds.

**Table 3:** Current treatment approaches for pediatric avoidant/restrictive food intake disorder: clinical role, evidence base, and key limitations.

Treatment Approach	Main Components and Clinical Role	Evidence Base	Key Limitations
Medical and nutritional management	Medical risk assessment; restoration of adequate intake; targeted correction of nutritional deficiencies; monitoring of growth, hydration, electrolytes, and refeeding risk; escalation of care when required	Clinical guidance, reviews, and inpatient pathway studies	Predominantly observational and guidance-based evidence; variable criteria for level of care
Dietetic strategies and oral supplementation	Accepted foods as a starting point; individualized dietary planning; gradual dietary expansion; food chaining; food fortification and oral nutritional supplements when indicated	Dietary reviews and indirect randomized-trial evidence from underweight children with picky eating	Limited direct ARFID-specific evidence; uncertain generalizability; oral nutritional supplementation alone does not address restricted dietary variety or the mechanisms maintaining avoidance
Feeding- and swallowing-skill interventions	Assessment and treatment of dysphagia, impaired chewing or oral-motor function, sensory-related feeding difficulties, and developmentally inappropriate feeding skills when present	Clinical reviews and consensus literature on ARFID–PFD overlap, with indirect evidence from pediatric feeding and autistic populations	Limited ARFID-specific evidence; heterogeneous interventions; applicable when feeding-skill dysfunction, dysphagia, sensory-related feeding difficulties, or co-occurring pediatric feeding disorder is present
CBT-AR	Psychoeducation; regularization of eating; structured exposure; reduction in avoidance and safety behaviors; presentation-specific interventions targeting the predominant mechanisms maintaining restriction	Treatment-model descriptions, preliminary uncontrolled and mechanism studies, and broader systematic-review evidence	Controlled and comparative evidence remains very limited; most available studies are uncontrolled, with limited evidence regarding long-term outcomes and generalizability across age groups and clinical presentations.
Fear-focused exposure-based interventions	Graduated food, situational, and interoceptive exposure adapted from anxiety- and panic-disorder treatment; reduction in safety behaviors; modification of catastrophic beliefs	Conceptual and treatment-model literature on fear-driven presentations	Limited ARFID-specific controlled evidence; nonstandardized methods and outcomes
Behavioral feeding interventions in autistic children and young people	Reinforcement, shaping, modeling, structured exposure, stimulus fading, environmental adaptation, and modification of challenging mealtime behaviors	Small and heterogeneous studies of food selectivity and feeding difficulties	Formal ARFID diagnosis often absent; evidence derives predominantly from younger children; limited generalizability and variable intervention components
Family-based and parent-focused interventions	Caregiver-supported nutritional rehabilitation; reduction in accommodation; support for exposure and dietary expansion; modification of caregiver responses to avoidance	Case studies and pilot studies; one randomized clinical trial (RCT) using telehealth showed a greater increase in estimated body weight as a percentage compared to individual psychoeducational motivational therapy, with no overall difference between groups in terms of ARFID symptom severity.	One RCT using FBT; underweight children aged 6–12 years; limited generalizability
Pharmacotherapy	Adjunctive, off-label treatment of comorbid symptoms or selected clinical targets; examples include mirtazapine, SSRIs, and olanzapine	Guidelines, reviews, case reports, case series, and retrospective studies	No established efficacy for ARFID itself; no randomized pharmacotherapy trials; low-certainty evidence, frequent polypharmacy, and concurrent nonpharmacological treatment

The evidence categories presented in this table are descriptive and do not constitute a formal GRADE assessment. Treatment selection should be individualized according to the predominant maintaining mechanisms, developmental stage, medical and nutritional risk, feeding and swallowing skill profile, comorbidities, patient and family preferences, and availability of specialist services. Abbreviations: ARFID, avoidant/restrictive food intake disorder; CBT-AR, cognitive-behavioral therapy for ARFID; FBT-ARFID, family-based treatment for ARFID; PFD, pediatric feeding disorder; SSRI, selective serotonin reuptake inhibitor.

## Data Availability

No new data were created or analyzed in this study. Data sharing is not applicable to this article.
